# Neuroinflammation and Cerebrovascular Disease in Old Age: A Translational Medicine Perspective

**DOI:** 10.4061/2011/857484

**Published:** 2011-10-31

**Authors:** Mario Di Napoli, Imtiaz M. Shah

**Affiliations:** ^1^Neurological Service, San Camillo de'Lellis General Hospital, 02100 Rieti, Italy; ^2^Neurological Section, SMDN-Center for Cardiovascular Medicine and Cerebrovascular Disease Prevention, 67039 Sulmona (AQ), Italy; ^3^Strathclyde Institute of Pharmacy and Biomedical Sciences, University of Strathclyde, Glasgow, Scotland G4 0RE, UK

## Abstract

The incidence of cerebrovascular disease is highest in the elderly population. However, the pathophysiological mechanisms of brain response to cerebral ischemia in old age are currently poorly understood. Ischemic changes in the commonly used young animal stroke models do not reflect the molecular changes associated with the aged brain. Neuroinflammation and oxidative stress are important pathogenic processes occurring during the acute phase of cerebral ischemia. Free radical generation is also implicated in the aging process, and the combination of these effects in elderly stroke patients could explain the higher risk of morbidity and mortality. A better understanding of stroke pathophysiology in the elderly patient would assist in the development of new therapeutic strategies for this vulnerable age group. With the increasing use of reperfusion therapies, inflammatory pathways and oxidative stress remain attractive therapeutic targets for the development of adjuvant neuroprotective agents. This paper will discuss these molecular aspects of acute stroke and senescence from a bench-to-bedside research perspective.

## 1. Introduction

Old age is an important risk factor for stroke and is associated with increased patient morbidity and mortality [1, 2]. Many of these patients have associated comorbidities, for example, cardiovascular and respiratory disease. This is further complicated by an increased risk of cognitive and functional decline in elderly stroke patients [3, 4]. Poor functional recovery has also been demonstrated in aged-animal models [5]. The pathophysiological mechanisms of the brains response to an ischemic insult in old age are poorly understood. Most preclinical stroke studies have been performed in young animal models and therefore do not reflect the molecular changes associated with the aged brain [6, 7]. This has been one of the criticisms of preclinical stroke neuroprotection studies and implicated in the resulting failure of clinical stroke neuroprotection trials [8, 9]. 

Neuroprotective therapies targeting NMDA and AMPA receptors have demonstrated reduced efficacy in aged-animal stroke models [10]. The pharmacokinetic and pharmacodynamic properties of neuroprotective agents may also be different in older patients [8]. This therefore emphasizes the importance of assessing potential neuroprotective therapies in preclinical aged animal stroke models and early clinical studies of elderly patients [6]. A better understanding of stroke pathogenesis in the aged brain would assist in the development of new therapeutic strategies for treatment of this vulnerable age group [5, 11].

Acute ischemic stroke triggers an inflammatory cascade which causes injury to the cerebral tissue, and this process can continue for several days. Cerebral ischemia results in the generation of reactive oxygen species (ROS), which induce the expression of inflammatory cytokines and chemokines. Cytokines upregulate the expression of cell adhesion molecules, which leads to leukocyte infiltration of the cerebral infarct. Cytokines also activate resident microglia, which leads to increased oxidative stress and the release of matrix metalloproteinases. These postischemic molecular changes lead to dysfunction of the blood-brain barrier (BBB), cerebral edema, and neuronal cell death [12]. The secondary inflammatory response associated with acute stroke has been shown to worsen clinical outcome and results in increased cerebral infarct size [13–15]. Inflammatory mediators and oxidative stress are also implicated in reperfusion injury after thrombolysis and mechanical embolectomy, which can result in further neuronal injury [16, 17]. Furthermore, injury to the brain can make the body more vulnerable to systemic infections. A central nervous system injury-induced immunodepression syndrome has been identified in experimental stroke models leading to spontaneous systemic bacterial infections within 3 days after stroke [18, 19]. This suggests that early administration of potential neuroprotective therapies (within the first 6 hours) would be the optimal time for modifying the neuroinflammatory response. 

Therapeutic targeting of the neuroinflammatory pathways has therefore become an important area of translational medicine research in acute stroke [16, 17, 20]. The generation of free radicals and increased oxidative stress is also implicated in the aging process, and the combination of these effects in elderly stroke patients could explain the higher risk of morbidity and mortality [6, 21]. This paper will discuss the neuroinflammatory aspects of acute ischemic stroke and senescence from a translational medicine research perspective.

## 2. Inflammatory Mediators in Acute Stroke

**Figure 1:**
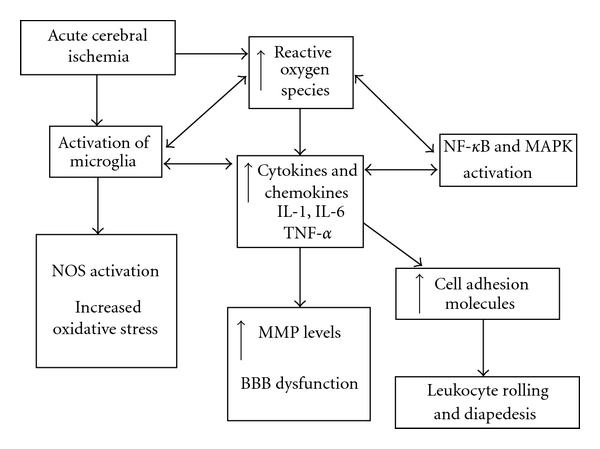
Acute cerebral ischemia and neuroinflammation. Acute stroke triggers an inflammatory cascade via the activation of a number of molecular mediators. The initial phase is associated with the generation of reactive oxygen species (ROS) within the ischaemic cerebral tissue. This is followed by the release of inflammatory cytokines and chemokines, which subsequently results in activation of resident microglia and upregulation of cell adhesion molecules (CAMs). The chemokines are involved in the mobilisation of leukocytes, and these inflammatory cells then interact with the CAMs. This leads to leukocyte infiltration of the ischaemic tissue (diapedesis), which further exacerbates the inflammatory process. Activation of nuclear factor kappa-B (NF-*κ*B) and inducible nitric oxide synthase (iNOS) results in increased oxidative stress and further cytokine activation. Release of matrix metalloproteinases (MMPs) from astrocytes and microglia leads to blood-brain barrier (BBB) dysfunction, cerebral oedema, and neuronal cell death. The aging process further exacerbates these neuroinflammatory pathways, and this has been associated with increased cognitive decline and poor functional outcome in elderly stroke patients. Therapeutic targeting of these molecular pathways is an important area of translational medicine research in cerebrovascular disease.

The cytokines and chemokines are important inflammatory mediators which are upregulated within the cerebral tissue during the acute phase of stroke (Figure 1). As well as being expressed by cells of the immune system, cytokines are also produced endogenously by the resident brain cells (microglia and neurons). Cytokines possess both pro- and anti-inflammatory properties, which play an important role in the progression of the cerebral infarct [22–24]. However, the spatial and temporal upregulation of cytokines and their receptors depends on the ischemic model used [25]. The main cytokines involved in neuroinflammation are the interleukins (IL), IL-1, IL-6, IL-10, and tumor necrosis factor-*α* (TNF-*α*). Cytokines are responsible for the initiation and regulation of the inflammatory response and play an important role in leukocyte infiltration into the ischemic regions of the brain [26–30]. The chemokines, monocyte chemoattractant protein-1 (MCP-1), and cytokine-induced neutrophil chemoattractant (CINC) also play an important role in cerebral ischemia and are potent chemoattractant factors [31]. In aged rat models of stroke, it has been demonstrated that the cerebral infarct develops more rapidly after reversible ischemia, with increased microglial response and cytokine expression [5, 32, 33]. This results in accelerated scar tissue formation and is associated with poor functional recovery [5]. Exacerbation of cerebral injury, via increased microglial activation, has also been demonstrated in aged-animal models of intracerebral hemorrhage [34]. The cytokine response has also been demonstrated to increase the risk of neurodegeneration and cognitive decline in aged animal models [35, 36]. Cytokines are also implicated in age-related cerebral atrophy, and an acute-on-chronic cerebral insult is likely to further exacerbate cognitive decline in old age [37].

## 3. Cytokine Activation in Acute Stroke and Senescence

The interleukins (IL-1, IL-6) and TNF-*α* have been the best-studied cytokines in the pathogenesis of acute stroke. These inflammatory mediators have also been implicated in the aging process [38–40]. 

### 3.1. Interleukin-1

The interleukin-1 (IL-1) family consists of the agonistic isoforms IL-1*α* and IL-1*β*, and their endogenous inhibitor, the IL-1 receptor antagonist (IL-1ra) [41]. The expression of IL-1*β* mRNA is rapidly observed after permanent middle cerebral artery occlusion (MCAo) and remains persistent for several days [42]. The important role of IL-1*β* in the pathogenesis of cerebral injury after stroke has been demonstrated by treatment with IL-1ra, which decreases neuronal cell death in the penumbral tissue and reduces infarct size after permanent focal cerebral ischemia [43]. The temporal induction profile of IL-1ra after ischemia virtually parallels that of IL-1*β* which may suggest that the balance between IL-1*β* and IL-1ra is more important than the levels of IL-1*β* itself [44].

### 3.2. IL-1ra in the Treatment of Acute Stroke

The phase II clinical trial of recombinant human IL-1ra (rhIL-1ra) (Anakinra or Kineret) demonstrated that patients with cortical infarcts in the treatment group had a more favorable clinical outcome (Table 1) [45]. The white cell count and inflammatory marker levels were also found to be lower in the treatment group. There is ongoing research into rhIL-1ra, as a potential neuroprotective agent in acute ischemic stroke [46]. A dose-ranging study has been performed in stroke patients to assess if Anakinra can easily cross the BBB and reach effective concentrations when administered intravenously. The results were favorable and showed that IL-1ra can enter the CSF and that the rate of entry can be modulated by altering the administration regime [47]. If the optimal therapeutic window can now be determined in acute stroke patients, this agent might be a promising and effective neuroprotective agent.

### 3.3. Interleukin-6

Interleukin-6 (IL-6) is a proinflammatory cytokine, which is secreted by monocytes in response to cerebral injury. Elevated levels of IL-6 in acute stroke patients correlate with a larger infarct volume and poorer clinical outcome [15, 58]. Increased IL-6 levels are also associated with senescence and frailty in old age [38]. This may further exacerbate stroke evolution in elderly patients, and this association of IL-6 with senescence requires further investigation [32, 35]. However, the role of IL-6 in acute stroke is far from clear as different regulatory levels have been demonstrated in experimental studies [59]. On one hand, IL-6 regulates synthesis and expression of acute-phase reactants, but it also possesses anti-inflammatory effects, which have been shown to be neuroprotective in both *in vitro *and *in vivo *studies [60, 61]. The effects of IL-6 during the different stages of acute stroke and genetic variation may assist in selective therapeutic targeting of this cytokine [62, 63]. Interestingly, increased cytokine activity has also been demonstrated in the muscles of the paretic limb after-stroke, and this may further hinder recovery [64]. This enhanced inflammatory state in elderly stroke patients may explain the increased risk of morbidity and mortality in this age group [65].

### 3.4. Tumor Necrosis Factor-*α*

Increased expression of TNF-*α* has been demonstrated in experimentally induced stroke models [66]. The initial source of TNF-*α* within the ischemic tissue appears to be from the microglia and macrophages although it has also been found in ischemic neurons [66, 67]. However, it is important to make a distinction between soluble and membrane-bound TNF-*α* [68]. Activated microglia and macrophages are major producers of soluble TNF-*α* within the first 6 hours after cerebral ischemia [69]. [70] TNF-*α* may show higher production rates in certain regions (e.g., striatum). Transient MCAo animal models and clinical stroke studies have also demonstrated increased peripheral TNF-*α* levels [22]. Increased levels of TNF-*α* have also been associated with senescence and neurodegeneration [36]. Intracerebral administration of TNF-*α*, 24 hours prior to MCAo, significantly enlarges infarct size, and treatment with anti-TNF-*α* antibodies has shown a reduction in infarct size [66, 71]. Therapeutic targeting of the TNF-*α* converting enzyme (TACE) is also being explored as a potential method of reducing TNF-*α* expression in acute stroke [72]. However, as with IL-6, TNF-*α* has also demonstrated neuroprotective effects in cerebral injury and could be related to the different stages of stroke pathogenesis [73]. Perhaps most importantly, TNF-*α* activates the NF-*κ*B pathway that is involved in signaling cell death (apoptosis) as well as cell survival. NF-*κ*B will stimulate the production of proinflammatory cytokines [74]. Ultimately, the balance between the two signals will determine the toxic degree of TNF-*α* [75]. Several hypotheses exist, one suggests that the detrimental effects occur in the early acute phase of the inflammatory response and the more beneficial effects in a later subacute stage.

### 3.5. TNF-*α* and Neuroprotective Effects in Ischemic Stroke

TNF-*α* has demonstrated beneficial effects in ischemic preconditioning when animal models were treated with lipopolysaccharide prior to MCAo [73]. Ischemic preconditioning is a procedure whereby brief episodes of ischemia are protective against a subsequent, more severe insult [76]. One factor that may mediate the neuroprotective effect of ischemic preconditioning is inflammation [77, 78]. Preconditioning with low doses of the proinflammatory agent lipopolysaccharide (LPS) in the rat provides a delayed tolerance and neuroprotection against subsequent challenge via focal ischemia in the brain [79, 80]. Likewise, a mild systemic inflammation elicited prior to stroke in a rat model for periodontitis has a neuroprotective effect by reducing the infarct volume in a rat model for cerebral ischemia [81]. It was hypothesized that the reduction in the infarct volume was due to a reduction in the number of macrophage-like cells that when present cause an enlargement of the infarcted area [81]. Two mechanisms have been put forward to explain the neuroprotective effect of proinflammation; one that depends on inducible NO synthase and peroxynitrite [79, 82], and a second one hypothesizing that LPS preconditioning suppresses neutrophil infiltration into the brain and microglia/macrophage activation in the ischemic hemisphere, which is paralleled by suppressed monocyte activation in the peripheral blood [83]. However, ischemic preconditioning via previous transient ischemic attacks (TIAs) does not seem to have a neuroprotective effect in elderly stroke patients, and these effects require further investigation [84]. Both interleukins and TNF-*α* are responsible for the activation of inducible nitric oxide synthase (iNOS), which produces nitric oxide (NO) and cyclo-oxygenase 2 (COX-2), a free radical producing enzyme [85]. This increased oxidative stress further worsens neuronal injury and is also related to the aging process [86]. Selective therapeutic targeting of these cytokines during the acute phase of stroke may potentially improve functional recovery. In conclusion, there is no consensus on the effect of TNF-*α* after ischemic stroke. Neurotoxic or neuroprotective effects will depend on several factors such as the extent of microglial activation in specific brain regions, timing, and threshold of TNF-*α* expression and of its receptors, and on the conditions that stimulate TNF-*α* signaling [87, 88]. It is also important to know which form of TNF-*α* is induced, in which cells, and on which receptor it will exert its effect.

### 3.6. Interleukin-10

Interleukin-10 (IL-10) is an anti-inflammatory cytokine which inhibits both IL-1*β* and TNF-*α* [89]. It has been demonstrated to reduce cerebrovascular risk in clinical studies of ischemic stroke [90]. IL-10 regulates a variety of signaling pathways and promotes neuronal and glial cell survival by blocking the effects of proapoptotic cytokines, as well as promoting expression of cell-survival signals [89]. As IL-10 has been shown to be an anti-inflammatory cytokine, exogenous administration of this cytokine could be a possible therapeutic strategy to reduce cerebral injury after stroke.

### 3.7. Transforming Growth Factor-*β*

Transforming growth factor-*β* (TGF-*β*) is another anti-inflammatory cytokine and is present within microglia [91]. Animal models of stroke have demonstrated neuroprotective effects of TGF-*β* in cerebral ischemia [92]. It is mainly expressed during the recovery phase of stroke and may contribute to cerebral remodeling via fibrosis and scar formation. More specifically after stroke, TGF-*β* reduces glial activation, decreases the expression and efficacy of other cytokines, and suppresses the release of harmful oxygen and nitrogen-derived products. TGF-*β* has been shown to reduce infarct volume by attenuating chemokine expression in the ischemic brain of animal models [93]. However, as TGF-*β* can inhibit apoptosis of neurons, but not necrosis, its possible protective influence is consequently limited to the penumbra. TGF-*β* could therefore be neuroprotective by blocking apoptotic pathways in the ischemic penumbra and aiding recovery of reversible ischemic brain tissue [94]. On the other hand, TGF-*β* stimulates glial scar formation and production of beta amyloid precursor, which can lead to a higher risk of cognitive deficit.

### 3.8. Granulocyte-Colony Stimulating Factor (G-CSF)

The cytokine growth factor, granulocyte-colony stimulating factor (G-CSF) has also shown some beneficial effects in aged animal stroke models, possibly via neurogenesis [95]. G-CSF promotes leptomeningeal collateral growth after common carotid artery occlusion and increases circulating blood monocytes and Mac-2-positive cells suggesting mechanisms coupled to monocyte upregulation [96]. Moreover, G-CSF stimulates neuronal differentiation of adult neural stem cells in the brain and improves long-term recovery in more chronic stroke models. G-CSF is being further evaluated in clinical stroke studies [97, 98]. 

These anti-inflammatory cytokines could therefore play an important role in potential neurorestorative and neuroregenerative therapies [99]. However, in aged animal models, there is accelerated gliosis and scar formation after-stroke, which could reduce the efficacy of these new therapeutic approaches [5, 100]. TGF-*β* signaling has been shown to increase in aged animal models of stroke via increased activity of microglia and astrocytes [101]. The effects of aging and anti-inflammatory cytokine expression therefore require further research in stroke pathogenesis and investigation of potential new treatments in aged animal models [6, 11].

## 4. Chemokine Expression in Acute Stroke and Effects of Aging

The chemokines are chemotactic cytokines, which mediate both leukocyte migration and microglial activation. There are 40 different known chemokines thus far, which all share a common structural pattern with 4 cysteine residues, which leads to their classification into four subfamilies of which two have an important role in stroke pathogenesis: the C-X-C and C-C family [102]. The C-X-C family attracts neutrophils and the C-C family monocytes/macrophages [103]. These are extensively expressed after cerebral ischemia [103, 104]. IL-6 and TNF-*α* both regulate the expression of MCP-1 and CINC within the cerebral tissue [104, 105]. Animal and cell culture studies have also demonstrated that both MCP-1 and CINC play an important role in ischemia-induced inflammatory response and cerebral tissue damage [103–105]. These studies indicate that CINC release precedes neutrophil accumulation and that MCP-1 plays a significant role in the migration of macrophages into the penumbral zone during cerebral ischemia. Increased MCP-1 levels have also been associated with aging and increased risk of neurodegeneration in animal studies [106].

The maximal expression of MCP-1 has been observed within 48 hours after-cerebral ischemia [31]. The different temporal production of MCP-1 and CINC contributes to the regulation of infiltrated leukocytes and the inhibition of MCP-1 and CINC signalling [103]. These chemokines are also implicated in BBB dysfunction, which has been demonstrated to occur earlier in aged rat stroke models [107, 108]. This is associated with accelerated gliosis and scar tissue formation in aged animals [5, 100]. Modification of this response may improve functional recovery and efficacy of potential new neuroregenerative therapies. 

### 4.1. Interleukin-8

IL-8 is also classed as a chemokine (CXCL8) and is thought to contribute to tissue damage by activating neutrophil infiltration [109, 110]. Anti-IL-8 antibody was shown to significantly reduce brain oedema and infarct size [110]. IL-8 has also been shown to be associated with cerebral atrophy in the aging brain [37]. These chemokines could therefore be attractive targets for potential neuroprotective treatments in acute ischemic stroke [110, 111]. Therapy with a broad spectrum pan-chemokine inhibitor, given at the onset of reperfusion, can reduce infarct volume by 50% after a 1-hour MCAo in rats [112]. These animals showed less macrophage accumulation in the peri-infarct area, but not in the core of the insult. This supports the hypothesis that inflammatory cells contribute to extended damage within the ischemic penumbra.

## 5. Early Gene Expression in Acute Stroke

Cerebral ischemia and the resulting increased oxidative stress activate early gene expression. This plays an important role in the neuroinflammatory response after cerebral injury and results in the production of cytokines, acute phase proteins, and other inflammatory mediators [113]. Microarray studies have demonstrated that over 400 genes could be activated during cerebral ischemia [113, 114]. One of the important transcription factors implicated in the inflammatory cascade is nuclear factor kappa-B (NF-*κ*B). This is a major mediator in the brain's response to ischemia and reperfusion and in the pathogenesis of acute stroke [30, 115]. NF-*κ*B activation has also been associated with age-related neurodegeneration [116]. NF-*κ*B is activated by a number of factors that are present during cerebral ischemia, which include activated glutamate receptors, reactive oxygen species (ROS), TNF-*α*, and IL-1*β* [115, 117]. NF-*κ*B is an important regulator of the inflammatory cascade, and many inflammatory mediators such as inflammatory cytokines, cell adhesion molecules (CAMs), and iNOS have NF-*κ*B binding sequences in their promoter regions [118]. It has several different targets and effects in various cell types and tissues, which can appear paradoxical [119, 120]. In some studies, preventing NF-*κ*B activation was shown to be protective, whereas in other studies, activation of NF-*κ*B enhanced neuronal survival [119–121]. These conflicting results may be due to the fact that NF-*κ*B can upregulate both proinflammatory and prosurvival factors that act in different ways depending on cell subtype [121]. 

Therapeutic targeting of the NF-*κ*B pathway has therefore become an attractive treatment option, as a central target of the neuroinflammatory cascade. Proteasome inhibitors have shown promising results in animal models of acute stroke [122]. NF-*κ*B, in its inactive form, is normally complexed to the inhibitory protein, inhibitory *κ*B (I*κ*B). Phosphorylation of I*κ*B by I*κ*B kinase (IKK) leads to activation of NF-*κ*B. Phosphorylated I*κ*B is then ubiquitinated and subsequently undergoes degradation by the proteasome. Proteasome inhibition has become an attractive target for drug discovery research in an attempt to reduce NF-*κ*B activation by preventing I*κ*B degradation, thus resulting in a dampening of the inflammatory response [123]. Hypothermia has been also shown to attenuate NF-*κ*B transcriptional activity in aged rat models of stroke, and the use of hypothermia in acute stroke is currently being explored in clinical trials [124, 125]. However, NF-*κ*B activity may be beneficial during the recovery phase of stroke and involved in cerebral remodelling [121]. The effects of cerebral ischemia and senescence can also result in proteasomal dysfunction, which can progress to cell death [126, 127]. Therefore, careful evaluation of potential drugs targeting the ubiquitin-proteasome system and NF-*κ*B is required.

## 6. Sirtuins and Telomerase Effects on Aging and Neuroprotection

Sirtuin proteins have been associated with longevity and influence aging by regulation of transcription and apoptotic factors [128]. Humans possess seven sirtuin proteins with sirtuin1 (Sirt1) being the best studied. Sirt1 is a deacetylase enzyme and influences inflammatory pathways via regulation of NF-*κ*B activity [129]. The flavonol compound, icariin, has demonstrated antioxidant activity and neuroprotection via upregulation of Sirt1 [130]. Resveratrol, a chemical found in red wine, has been shown to increase activity of Sirt1 and attenuate inflammatory response in animal models of stroke [131]. It has also shown neuroprotective effects via ischemic preconditioning associated with Sirt1 activation [132]. Resveratrol is being further evaluated in clinical studies [133]. 

Telomeres are repetitive sequences of DNA, located at the end of chromosomes and have a protective role in preventing chromosomal damage. Telomere attrition is associated with senescence and exacerbated by oxidative stress [134]. This has been implicated in the pathogenesis of atherosclerosis and vascular disease [135]. The telomerase enzyme and its catalytic subunit telomerase reverse transcriptase (TERT) maintain telomere length during cell replication. TERT knockout animal models of stroke have demonstrated enhanced neuroinflammatory response and increased infarct volume [136]. Sirt1 has been shown to have a telomere protective effect by reducing oxidative stress and maintaining telomerase activity [137]. The effects of telomerase and Sirt1 are exciting new areas of translational stroke and aging research.

## 7. Effect of Heat Shock Proteins in Acute Stroke and Senescence

Heat shock proteins (HSPs) are molecular chaperones, which play an important role in cellular metabolism. They also have an important role in controlling the neuroinflammatory pathways [138]. HSP70 is the major HSP and is constitutively expressed in neuronal tissue [138]. Neuronal injury and increased oxidative stress induce HSP70 expression. Animal HSP70 knockout studies have demonstrated increased cerebral infarct size [139]. The HSPs also reduce NF-*κ*B activation in animal models of cerebral ischemia by interfering with I-*κ*B phosphorylation by IKK [138]. This attenuates the neuroinflammatory process, and the neuroprotective effects of HSPs are being further investigated for potential treatment in acute stroke [140]. The HSPs have also demonstrated protective effects from aging and associated with human longevity [141]. Chaperonotherapies could therefore be an attractive therapeutic option for neuroinflammatory disease treatment in old age [142, 143]. 

## 8. Cell Adhesion Molecules in Acute Stroke Pathogenesis

The accumulation and infiltration of the cerebral tissue by leukocytes is a complex process that requires the interaction between several cell adhesion molecules (CAMs) and chemokines [144, 145]. The leukocytes roll on the endothelial surface and then adhere to the endothelial cells, which leads to diapedesis (Figure 1). The rolling of leukocytes is mediated by interaction of E- and P-selectin (found on the surface of endothelial cells), and L-selectin (normally found on the surface of leukocytes) with their respective ligands [144]. Inhibiting the activity of P-selectin alone by treatment with monoclonal antibodies (ARP 2–4, RMP-1) after the onset of the insult does not reduce the infarct volume significantly [146–148]. This suggests that the involvement of P-selectin in the inflammatory response after ischemic injury starts early. Firm adhesion and activation of leukocytes is mediated by binding of the CD11/CD18 complex to CAMs, such as intercellular cell adhesion molecule-1 (ICAM-1), vascular cell adhesion molecule-1 (VCAM-1), platelet-endothelial cell adhesion molecule-1 (PECAM-1), and the mucosal addressin [117, 144, 149]. Increased circulating levels of CAMs have also been associated with aging, which could exacerbate leukocyte infiltration [150]. IL-6 and TNF-*α* also regulate the expression of CAMs on the endothelial cells and induce infiltration of the ischemic penumbra by leukocytes at the site of inflammation [144]. There is ample evidence from animal models of MCAo that expression of CAMs is associated with increased cerebral infarct size [144]. When MCAo in experimental stroke was followed by reperfusion, administration of anti-CAM antibodies decreased infarct size [151]. However, anti-CAM treatment has not been successful in clinical studies of acute ischemic stroke. The enlimomab study used a monoclonal antibody against ICAM-1, which was administered within 6 hours of acute ischemic stroke onset (Table 1). The three-month outcome mortality data and adverse events were worse in the enlimomab group, and it appears that there may have been a proinflammatory response [28]. Surprisingly, for PECAM-1, there is suggestion of possible neuroprotective properties aside from the neurotoxic effects. PECAM-1 knock-out mice show facilitated leukocyte transendothelial migration after histamine treatment which contradicts the current hypothesis that PECAM-1 stimulates migration [149]. Further research into therapeutic targeting of CAM is ongoing [152, 153].

## 9. Inflammatory Cells in Acute Stroke

Neuroinflammation is characterized by an accumulation of inflammatory cells and chemical mediators within the cerebral infarct (Figure 1). Neutrophil infiltration in acute stroke has been demonstrated by single photon emission computer tomography (SPECT) studies [154]. This is associated with increased cerebral infarct size [26]. A significant inflammatory response has also been demonstrated in aged animal models of stroke [33, 155]. Clinical studies have shown that peripheral inflammatory cells also play an important role in the pathogenesis of cerebral ischemia [26]. This has also been demonstrated in numerous animal models of acute stroke [92]. MRI animal studies have shown neutrophil infiltration into the infarct zone within a few hours of ischemia, and this process peaks at 24 hours [156]. Blood-derived macrophages and activated microglial cells contribute to the postischemic brain damage by expressing iNOS and production of cytotoxic agents and ROS [30]. Macrophage activation has been shown to exacerbate cerebral injury in aged animals [34]. How microglia become activated after ischemic stroke is still not clear. A possible mechanism is rupture of necrotic neurons in the core of the insult, leading to release of their contents into the extracellular space and scavenging of these contents by microglia [30]. Microglia/macrophages surrounding the ischemic tissue will migrate toward the ischemic lesion and engage in close contact with neurons (“capping”). As these neurons die later on, this capping ensures early recognition and fast phagocytic removal of dying/dead neurons [157]. In an activated state, microglia will produce inflammatory and cytotoxic mediators contributing to cell damage and cell death. On the other hand, microglia are a major producer of TGF-*β*1 which supports the hypothesis that microglial activation is also neuroprotective [25]. Finally, resident macrophages scavenge and remove necrotic debris and harmful components. Indeed, these data suggest that early activation is detrimental and later activation beneficial. Different subsets of microglia may have different roles after ischemic stroke and thus improve or reduce the chances of survival of ischemic neurons [158, 159]. Perhaps an ideal therapy should modulate the microglial response in order to stimulate neurogenesis [160]. Transgenic mice in which microglial proliferation can be inhibited (eliminated) show increased infarct volume (by 13%) after a 1-hour occlusion, which suggests that proliferating resident microglial cells exert a neuroprotective role after ischemia [161]. Microglial activation has also been associated with stimulation of the toll-like receptor 4 (TLR4). Permanent MCAo models of TLR4-deficient mice were shown to have reduced infarct size [162]. TLR4 plays an important role in the initiation of the inflammatory response during cerebral ischemia and has become another important target for neuroprotective therapy. However, TLR activity seems to decline with aging, and further investigation into its role in cerebrovascular disease is required [163]. 

Inhibition of leukocyte activation and infiltration into the ischemic cerebral tissue have therefore been an important area of neuroprotection drug research [152, 164]. The neutrophil inhibitory factor, UK-279, 276, a recombinant protein inhibitor of the CD11/CD18 receptor, demonstrated reduced infarct size in animal models of stroke. However, the Acute Stroke Therapy by Inhibition of Neutrophil (ASTIN) study did not show any patient benefit and was terminated for futility (Table 1) [48]. Neuroimaging studies have demonstrated inflammatory cell infiltration during acute stroke and identification of salvageable ischemic penumbra [165]. These imaging techniques can be used as important surrogate markers for future anti-inflammatory drug studies. The use of both neuroradiological markers and biomarkers as surrogate measures of drug treatment in clinical stroke trials is an important Stroke Treatment Academic Industry Roundtable (STAIR) recommendation [8].

## 10. Free Radicals and Increased Oxidative Stress

Increased free radical production and oxidative stress play an important role in the pathogenesis of both acute stroke and senescence [21, 166]. Activated microglia/macrophages further increase free radical generation and exacerbate cerebral injury in aged-animal models of stroke [34]. This is associated with mitochondrial dysfunction and results in neuronal cell death [167]. Nitric oxide (NO) is one of the important reactive oxygen species (ROS). It possesses both neuroprotective and neurotoxic properties in cerebral ischemia. This is related to the activation of the three different isoforms of NO synthase (NOS) at different stages of the ischemic process. The three isoforms of NOS are endothelial (eNOS), neuronal (nNOS), and inducible (iNOS). NOS catalyses the chemical conversion of L-Arginine to NO and citrulline. The constitutive isoforms (eNOS and nNOS) are activated by increased levels of intracellular calcium, during the acute phase of cerebral ischemia. Neuronal NOS has a much higher capacity for NO generation than eNOS, and this is responsible for neuronal damage during the early stages of ischemic stroke. Inducible NOS activation occurs later, usually 12–48 hrs after the initial ischemic insult [85]. This is associated with a much higher production of NO, and for a longer period, compared to its two isoforms. 

The effect of eNOS is well known for its vasodilatory properties, via the action of cyclic GMP. However, the activity of eNOS has been shown to diminish in aged-animal models [168]. Studies using eNOS knock-out mice have demonstrated increased infarct size following transient MCAo [169]. The upregulation of eNOS via the action of statins has provided an additional neuroprotective property to this class of lipid-lowering drugs [99]. On the contrary, nNOS knock-out mice have been shown to develop smaller infarct volumes in MCAo [170]. There is a strong association between the activation of NMDA receptors and calcium-dependent increase in nNOS activity. The toxic ROS, peroxynitrite (ONOO ^−^) produced from NO reactions has been associated with neuronal cell death, via lipid peroxidation and DNA damage [30, 171]. Increased expression of proapoptotic proteins in aged animal stroke models, via constitutive NOS activation, has also been associated with neuronal cell death [172]. Inducible NOS upregulation results in further NO generation during the later stages of cerebral ischemia [85]. Leucocytes and endothelial and glial cells are the main sources of iNOS expression. Selective inhibitors of iNOS have been shown to display neuroprotection for up to 5 days after-ischemic insult [85]. Again, peroxynitrite is the main ROS involved in neuronal cell death. Interactions between iNOS and cyclo-oxygenase 2 (COX-2) have been linked to penumbral cell death in late cerebral ischemia [85]. Due to this late and prolonged activation of iNOS, it remains an important therapeutic target for potential antioxidant and spin-trap agent therapy [173]. 

Another important source of ROS is NADPH oxidase. This enzyme predominantly produces the superoxide anion, which reacts with NO to generate peroxynitrite [166]. Recently, there has been increasing interest in the role hydrogen sulfide (H_2_S) in the pathogenesis of cerebral ischemia [174]. H_2_S is formed from the amino acid cysteine, via the action of the enzyme cystathionine beta-synthase. However, H_2_S has demonstrated both beneficial and detrimental effects during acute cerebral ischemia [175, 176]. Clinical studies have demonstrated worse neurological outcome in stroke patients with higher plasma cysteine levels, reflecting increased H_2_S activity [175]. However, H_2_S has also demonstrated antioxidant properties via free radical scavenging and inhibition of iNOS [176, 177]. This dual effect of H_2_S could be related to the different stages of cerebral ischemia, and more research is required to investigate any potential therapeutic benefit of targeting this molecular pathway [174].

Due to the destructive nature of ROS in cerebral ischemia, therapeutic interventions have been an important area of translational stroke research. The increased oxidative stress associated with aging may further exacerbate cerebral ischemia and result in poor functional recovery in elderly stroke patients [21, 167]. However, clinical studies using natural antioxidants (vitamin C and E) and B vitamins to lower homocysteine have not shown any benefit in reducing vascular risk [178, 179]. These vitamins also seem to have little effect on the aging process, possibly because of low antioxidant activity at the smaller recommended doses [180]. The SAINT II [Stroke-Acute Ischemic—NXY-059 (Cerovive) Treatment] study investigated the effect of the nitrone spin-trap agent, NXY-059, in patients presenting within 6 hours of acute stroke symptom onset [8]. This nitrone-derived free radical trapping agent was shown to be an effective neuroprotective agent in animal models of stroke, with a large therapeutic window for treatment [181]. Unfortunately, results from the phase III studies were negative, for both ischemic and hemorrhagic stroke, and further development of the drug has been abandoned (Table 1) [49]. However, lessons from the SAINT studies have allowed further revision of the STAIR criteria and will lead to more rigorous translational research studies in the future [9, 181]. Research is ongoing into antioxidant spin trap agents in the treatment of acute stroke and senescence [21, 173].

Recent clinical studies of antioxidant agents have been more encouraging and shown positive results in early clinical stroke trials [51, 53] (Table 1). Edaravone (Radicut), a free radical scavenger, is currently being investigated as an antioxidant agent in the treatment of acute stroke [182]. Edaravone was shown to reduce activation of NF-*κ*B and MMP-9 in animal models of rt-PA-related hemorrhage [183]. This drug was also shown to reduce oxidative neuronal damage [184]. Clinical studies have shown some benefits of edaravone in lacunar stroke patients with reduction in infarct size and early neurological recovery [51] (Table 1). Uric acid has also been shown to have antioxidant activity and neuroprotective effects in animal models of ischemic stroke [185]. Clinical studies have demonstrated reduced levels of uric acid during the acute phase of stroke, which may exacerbate oxidative stress. The use of adjuvant uric acid treatment with rt-PA was shown to reduce lipid peroxidation and may have beneficial effects in patient outcome [53] (Table 1). Further clinical trials of uric acid in acute stroke are being planned [54].

A viable alternative to conventional drug-based neuroprotective therapies is brain/body cooling, or hypothermia. This provides neuroprotection by reducing oxidative stress, DNA damage, and neuronal apoptosis [186, 187]. In animal studies of focal ischemia, short-term hypothermia consistently reduces infarct size. Nevertheless, efficient neuroprotection requires long-term regulated lowering of whole body temperature. Exposing poststroke-aged rats to a mixture of air and a mild inhibitor of oxidative phosphorylation, H_2_S, for 2 days, resulted in sustained, deep hypothermia (30.8 ± 0.7°C). Long-term hypothermia led to a 50% reduction in infarct size with a concomitant reduction in the number of phagocytic cells. At the transcription level, hypothermia caused a reduction in the mRNA coding for caspase 12, NF-*κ*B, and grp78 in the peri-infarcted region, suggesting an overall decrease in the transcriptional activity related to inflammation and apoptosis. Behaviorally, hypothermia was associated with better performance on tests that require complex sensorimotor skills, in the absence of obvious neurological deficits or physiological side effects, in aged rats [188].

Mild-to-moderate intraischemic hypothermia reduces ATP depletion, anoxic depolarization, glutamate release, and apoptosis, maintains BBB integrity, inhibits white matter injury, and blocks necrosis (if started during ischemia itself) [189]. The effects of hypothermia on inflammation show a more modulating response. However, hypothermia has been suggested to only delay neuronal damage rather than to provide permanent protection. Furthermore, a distinction has to be made between short and long cooling periods. It has been suggested that the disadvantages of delayed cooling could be overcome by performing a prolonged hypothermic protocol [190]. However, the modulating influence of hypothermia on neuroinflammation could also differ depending on these parameters and requires further investigation [191]. Depth and duration of cooling both influence outcomes in experimental and clinical settings and may make translation to the clinic difficult [192].

## 11. Blood-Brain Barrier Dysfunction

Cerebral ischemia is associated with the release of matrix metalloproteinases (MMPs), as part of the neuroinflammatory response. These proteases are involved in the breakdown of the microvascular basal lamina, which results in the disruption of the BBB [193]. These changes are most prominent in the core infarct, where neuronal damage is maximal. The gelatinases (MMP-2 and MMP-9) are the main MMPs involved in destruction of the basal lamina. MMP-2 is expressed constitutively in the CNS and is normally present within cerebral tissue. MMP-9 is normally absent, and this is the major MMP associated with neuroinflammation [194]. These enzymes are released from endothelium, glia, and infiltrating leukocytes. They target laminin, collagen IV, and fibronectin proteins, which are the major constituents of the basal lamina. This is associated with BBB dysfunction and leads to cerebral edema [12]. Reduced infarct size has been shown in rat models of stroke treated with MMP inhibitors, and also in MMP-9 knock-out mice studies [195, 196].

MMP-9 levels play an important role in the development of cerebral edema and hemorrhagic transformation of infarcted cerebral tissue [197]. Clinical studies have demonstrated a strong correlation between elevated plasma MMP-9 levels and risk of hemorrhagic transformation in the acute phase of ischemic stroke [197]. Elevated MMP-9 concentrations have also been shown to be a predictor of thrombolysis-related intracerebral hemorrhage in patients treated with tissue plasminogen activator (t-PA) for acute ischemic stroke [198]. The combination of MMP inhibitors with t-PA could be a future treatment option in reducing bleeding complications associated with thrombolytic therapy [173]. However, recent animal studies have demonstrated that MMP inhibitors could worsen longer-term neurological outcome, and further evaluation of these drugs in acute stroke is required [199]. Broad-spectrum inhibitors of MMPs, such as BB-94 and KB-R7785, administered after stroke onset, reduce damage after permanent MCAo in mice by 26% [200, 201]. Such treatments, however, cause serious side effects due to their low specificity and explain why these inhibitors have not been used in clinical practice [202]. On that account, more selective inhibitors or knock-out mice for MMP-2 or -9 have been explored. MMP-2 knock-out mice subjected to 2-hour occlusion show massive upregulation of MMP-9, and obviously no improvement could be observed [203]. A selective inhibitor for MMP-2 and -9 (SB-3CT) in mice subjected to a 2-hour MCAo reduces lesion size up to 6 hours after ischemia onset, and this inhibitor is well tolerated in animals [202].

BBB dysfunction has been identified as a major cause of cerebral injury in aged animal stroke models [52, 107]. After ischemia, subtle dynamical changes in BBB permeability occur and can be transient or permanent depending on the severity of the insult. The latter is characterized by endothelial swelling, astrocyte detachment, and blood vessel rupture in the ischemic area, while transient BBB disruption shows endothelial hyperpermeability to macromolecules in the peri-infarct area [204]. Transient BBB disruption shows a biphasic pattern with an initial opening 2-3 hours after the onset of the insult, while 24–48 hours after reperfusion a second opening occurs, leading to vasogenic edema and increased intracranial pressure [204]. Furthermore, production of proinflammatory cytokines and adhesion molecules will be stimulated. Such a disruption results in rapid but significant changes in the molecular relationship between astrocytes and the microvascular extracellular matrix, which has a feedback effect on the neurons they supply and protect [205]. BBB dysfunction in old age has been shown to be closely related to white matter lesions and lacunar infarction [206, 207]. Cerebral amyloid angiopathy is also associated with BBB disruption in old age and subsequent increased risk of intracerebral hemorrhage [208]. 

**Figure 2:**
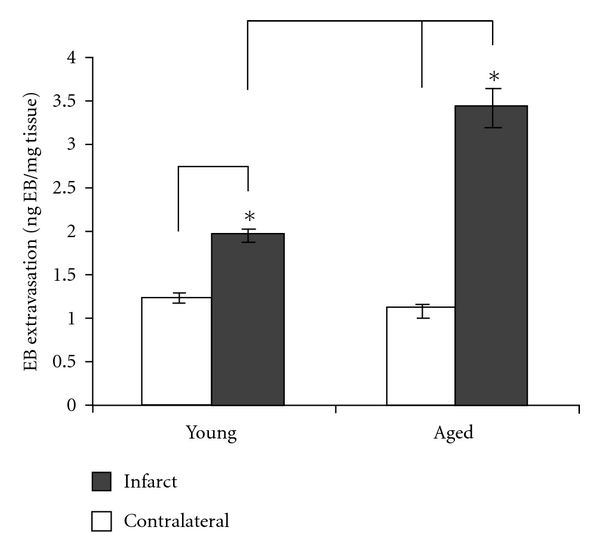
Extravasation of albumin across the BBB 20 min after-MCAO. The aged animals exhibited greater BBB permeability in relation to the corresponding young rats in the infarcted hemisphere (*P* < 0.001). (Copyright: DiNapoli et al. [107]).

BBB permeability studies have demonstrated minimal disruption within the first 6 hours after-MCAo but increased permeability by 21 hrs [209]. However, this BBB dysfunction occurs much earlier in aged rat models [107]. This was examined by comparing young (3 months) and aged (18 months) MCAo rat models. BBB disruption was assessed at 20 min and 24 hrs after-MCAo, with t-PA-induced reperfusion at 120 min. The results showed that BBB disruption in aged rats occurred earlier and increased nearly twofold at both 20 min and 24 hr time points compared to the younger animals (Figure 2) [107]. Neuronal damage in aged rats was also much worse compared to young rats at 24 hr, with aged rats suffering larger infarct size and reduced functional recovery. These findings suggest that early BBB disruption in aged stroke patients could contribute to a greater degree of neuronal injury. There have been concerns that t-PA treatment in elderly stroke patients may increase the risk of hemorrhagic complications, but recent clinical studies have shown no increased risk [210]. BBB dysfunction in old age is therefore an important area of translational stroke research [20, 210, 211]. The development of new MRI techniques for investigating BBB dysfunction in stroke will be an important investigational tool in future translational research [212].

## 12. Clinical Aspects of Neuroinflammation in Acute Stroke

In addition to the development of the localized inflammatory response in the brain, acute stroke also evokes an immune response at the systemic level [15, 213]. This is characterized by the release of proinflammatory mediators into the systemic circulation [14, 15]. The clinical manifestation is called the systemic inflammatory response syndrome (SIRS; Table 2). SIRS is evident in both ischemic and hemorrhagic stroke [214–216]. The degree of the inflammatory response has also been shown to be related to the size of infarct volume [13]. The inflammatory response is also associated with the development of hyperthermia during the acute phase of stroke [217]. This is related to stroke severity and associated with poor patient outcome [217, 218]. Animal stroke models have also demonstrated increased infarct size in hyperthermic conditions [219]. The neuroprotective effects of hypothermia in animal stroke models have demonstrated reduced activation of NF-*κ*B and inflammatory pathways [124]. Clinical research studies have also been investigating the neuroprotective effects of hypothermia, and further trials are planned [125, 220]. The effects of antipyretic treatment in hyperthermic acute stroke patients, as part of the Paracetamol (Acetaminophen) in Stroke (PAIS) trial, did not show any benefit in stroke patients, but post hoc analysis of temperature between 37 and 39°C did show improved patient outcomes with acetaminophen treatment [55] (Table 1). The role of prophylactic antibiotic use in acute stroke patients, in an attempt to treat associated infections and reduce inflammatory complications, is another area of ongoing research [221]. Treatment with minocycline, a bacteriostatic antibiotic with possible anti-inflammatory effects, has shown beneficial effects in a pilot study of acute stroke patients (Table 1), and further evaluation of this drug is ongoing [56, 57].

The neuroinflammatory response has also been associated with cognitive decline and delirium, which is frequently seen in elderly stroke patients [222, 223]. This seems to compromise the reduced functional reserve in the aged brain and is related to underlying cerebral small vessel disease [4]. An increased cerebral inflammatory response has also been demonstrated in aged animal studies with associated increased cognitive deficits [35]. Increased CRP levels have been associated with poststroke cognitive impairment in elderly patients [224]. The reduction of this systemic inflammatory response could potentially improve cognitive and functional outcomes in elderly stroke patients, and further research of these effects in aged stroke animal models is therefore important [181, 225]. With the increased risk of stroke in postmenopausal females, hormonal effects in stroke pathogenesis are also an important area of research. Many preclinical stroke studies have demonstrated neuroprotective and anti-inflammatory effects of estrogen [226]. However, hormonal replacement therapy in female patients has been associated with increased vascular risk in clinical studies [226]. Male sex is an important risk factor for stroke, but little is known about the effects of androgenic hormones in stroke outcome. Some studies have shown that high testosterone levels during the acute phase of stroke are associated with worse clinical outcome but may have a neuroprotective effect during the recovery phase [227]. However, clinical studies show increased vascular risk associated with testosterone supplementation in older men, but this may have some benefit in male patients with low testosterone levels [228]. These contradictory results in preclinical and clinical effects of sex hormone treatment therefore require further translational research.

C-reactive protein (CRP) is an indicator of underlying systemic inflammation. It is an acute-phase reactant and has a pronounced rise in concentration after tissue injury or inflammation. It does not seem to have a significant role in the aging process *per se* but more related to disease pathogenesis [229, 230]. CRP has a long plasma half-life and could also be a potential mediator as well as a marker of cerebrovascular disease [231]. The association of increased levels of CRP with ischemic stroke has been reported in several clinical studies [15, 232]. It has been shown that increased levels of CRP are associated with a worse outcome in patients with ischemic stroke [232, 233]. Increased levels of CRP are also associated with an increased risk of future stroke in elderly patients [90, 234]. However, the role of CRP in the pathogenesis of ischemic stroke is not completely understood. It is unclear whether CRP is just a marker of systemic inflammatory processes or directly involved in pathogenesis of cerebral tissue damage [231]. Further research to investigate any potential therapeutic benefits of inhibiting CRP in vascular disease is ongoing [235].

## 13. Conclusion

The incidence of stroke is highest in the elderly population, and the underlying pathogenic changes in combination with senescence have been associated with increased cerebral injury [11]. Acute cerebral ischemia results in a complex inflammatory cascade resulting in the activation of a variety of inflammatory cells and molecular mediators. Aged animal models have demonstrated a more intense inflammatory response during the acute phase of ischemia, followed by early scar formation and fibrosis. There is also earlier BBB dysfunction in older animals with increased permeability and neuronal injury. The neuroinflammatory response involves several molecular pathways, which are interconnected with the aging process and cognitive dysfunction. These complex molecular interactions in old age make it difficult to draw firm conclusions from research observations made in young animal models of stroke and translate these findings into clinical studies. The importance of conducting preclinical stroke studies in aged animal models will therefore be an important part of translational stroke research (STAIR criteria) in the future [6, 8].

 Numerous animal models of stroke have demonstrated reduced infarct size on modification of the inflammatory response although these inflammatory mediators also have beneficial effects during the recovery phase. Clinical studies have suggested that cerebral infarct size and patient outcome are affected by the inflammatory response. Unfortunately, clinical neuroprotective drug trials targeting the inflammatory pathways in acute ischemic stroke have thus far been disappointing. Most of the preclinical stroke studies have been performed in young animal models and therefore do not reflect the molecular changes associated with the aged brain. This has been one of the criticisms of preclinical stroke neuroprotection studies and implicated in the failure of clinical trials. A more rigorous bench-to-bedside research approach into investigating neuroprotective agents in a target elderly stroke population may allow a more successful transition into clinical trials and hopefully clinical practice [8, 181]. A multitarget approach together with reperfusion therapies may be the best therapeutic approach in the future.

There is sufficient data to support that hypothermia acts at multiple levels of the ischemic cascade and of the neuroinflammatory response. Hypothermia attenuates the expression of several inflammatory mediators at certain time points and appears to be an attractive therapeutic option, but more research is required to discern which positive or negative effects contribute to neuroprotection [236]. The ability of hypothermia to modulate many aspects of the inflammatory response may render translation to the clinic feasible [125]. Another advantage of hypothermia could be the creation of a larger therapeutic time window to administer other neuroprotective agents and thus improve outcome after transient focal cerebral ischemia. With the success of thrombolysis in the treatment of acute ischemic stroke and ongoing clinical trials of interventional reperfusion therapies, together with better MR imaging techniques, adjuvant neuroprotective therapies remain an attractive option in the future [27, 29].

##  Conflict of Interests

The authors report that they have no conflict of interests.

## Figures and Tables

**Table 1:** Neuroprotective agents targeting neuroinflammation in acute stroke.

Neuroprotective agent	Mode of action	Summary of clinical trials
Recombinant human IL-1 ra (rhIL-1ra)	Interleukin-1 receptor antagonist	In the phase II clinical trial of rhIL-1ra, patients within 6 hours of stroke symptom onset were randomised to either rhIL-1ra or placebo. In the rhIL-1ra-treated group, patients with cortical infarcts had a better clinical outcome [45].

Enlimomab	Anti-ICAM -1 monoclonal antibody	In the phase III clinical trial of enlimomab, patients were randomised to receive either the monoclonal antibody or placebo within 6 hours of acute stroke onset. The modified Rankin scale was worse in patients treated with enlimomab (*P* = 0.004), and treatment was associated with higher mortality. Further development of this drug has been abandoned [28].

UK-279, 276	Neutrophil inhibitory factor	In the Acute Stroke Therapy by Inhibition of Neutrophils (ASTIN) phase II clinical trial, patients were randomised to receive either an infusion of UK-279, 276, or placebo within 6 hours of acute stroke symptom onset. No efficacy was reported on administration of study medication, and the clinical trial was terminated for futility [48].

Cerovive (NXY-059)	Nitrone-based free radical trapping agent	In the phase III clinical trial, Stroke-Acute Ischemic—NXY-059 Treatment II (SAINT II) randomised patients within 6 hours of acute stroke onset to either an infusion of NXY-059 or placebo. There was no significant reduction in stroke-related disability, as assessed by the modified Rankin scale (*P* = 0.33). The Cerebral Hemorrhage And NXY-059 Treatment (CHANT) trial also showed no treatment effect on functional outcome. Further drug development has been abandoned [49, 50].

Edaravone (Radicut)	Free radical scavenger	Lacunar stroke patients treated with edaravone showed significant reduction in infarct size at 1-year followup and early improved neurological outcomes. There was no difference in overall clinical outcomes after 1 year [51, 52].

Uric acid	Antioxidant	The phase II double-blinded study investigated safety and pharmacokinetics of uric acid in acute stroke patients treated with rt-PA. Levels of uric acid increased in the treatment group, with reduction in lipid peroxidation. No safety concerns were reported with uric acid treatment. Further evaluation is ongoing [53, 54].

Acetaminophen (Paracetamol)	Antipyretic effect	In the Paracetamol (Acetaminophen) in Stroke (PAIS) clinical trial, patients presenting within 12 hours of acute stroke onset were randomised to either acetaminophen (6 g daily) or placebo for three days. There was no benefit seen for routine use of acetaminophen in acute stroke but post hoc analysis showed beneficial effects in patients with body temperature between 37 and 39°C [55].

Minocycline	Bacteriostatic antibiotic Anti-inflammatory effects	Stroke patients with NIHSS > 5 and symptom onset between 6 and 24 hours were randomised to either once daily minocycline 200 mg or placebo for 5 days. The NIHSS and modified Rankin scale were significantly lower in the treatment group at 90 days [56].
		The Minocycline to Improve Neurologic Outcome in Stroke (MINOS) study was a dose-escalation trial, administering intravenous minocycline within 6 hours of symptom onset. This was shown to be safe and well tolerated up to 10 mg/kg intravenous dosing [57].

**Table 2:** The systemic inflammatory response syndrome (SIRS).

SIRS-diagnostic criteria
SIRS diagnosed if 2 or more of following criteria are present:
Temperature >38°C or <36°C
Respiratory rate >20 breaths/min
Heart rate >90 bpm
White cell count >12,000 mm^3^ or <4,000 mm^3^ or >10% immature
neutrophils
